# Are there country-specific differences in the use of pegvisomant for acromegaly in clinical practice? An analysis from ACROSTUDY

**DOI:** 10.1007/s40618-022-01789-4

**Published:** 2022-03-31

**Authors:** S. Grottoli, A. Bianchi, F. Bogazzi, C. Bona, M. O. Carlsson, A. Colao, F. Dassie, A. Giampietro, R. Gomez, S. Granato, P. Maffei, R. Pivonello, N. Prencipe, M. Ragonese, C. Urbani, S. Cannavò

**Affiliations:** 1 Division of Endocrinology, Diabetology and Metabolism, Department of Medical Science, University of Turin, Corso Dogliotti 14, 10126 Turin, Italy; 2 Pituitary Unit, Department of Endocrinology, Fondazione A Gemelli, IRCCS, Università Cattolica del Sacro Cuore, Rome, Italy; 3 Department of Clinical and Experimental Medicine, University of Pisa, Pisa, Italy; 4 Global Medical Affairs, Pfizer Rare Disease, Brussels, Belgium; 5 Dipartimento di Medicina Clinica e Chirurgia, Sezione di Endocrinologia, Università Federico II di Napoli, 80131 Naples, Italy; 6 Department of Medicine, Padua University Hospital, Padua, Italy; 7 Medical Department, Pfizer Italia, Rome, Italy; 8 Unit of Endocrinology, Department of Human Pathology, University of Messina, Messina, Italy; 9 Endocrinology II Unit, Department of Medicine, Azienda Ospedaliero Universitaria Pisana, Pisa, Italy

**Keywords:** Acromegaly, Effectiveness, Pegvisomant, Real-world analysis, Safety, Treatment schemes

## Abstract

**Objective:**

A comprehensive picture of pegvisomant use for treating acromegaly in routine clinical practice in different countries is lacking. We aimed, therefore, to document country-specific behaviors in real-life pegvisomant use, and the main safety and effectiveness outcomes in the ACROSTUDY.

**Design:**

ACROSTUDY is an open-label, non-interventional, post-marketing safety surveillance study.

**Methods:**

A descriptive analysis was performed using data from the six top-recruiter ACROSTUDY countries, i.e., Germany (*n* = 548 patients), Italy (*n* = 466), France (*n* = 312), USA (*n* = 207), Spain (*n* = 200) and the Netherlands (*n* = 175). These nations accounted for > 85% of the ACROSTUDY cases.

**Results:**

The mean pegvisomant dose at treatment start was lowest in the Netherlands (9.4 mg/day), whereas it ranged between 10.9 and 12.6 mg/day in the other countries. At year 5, the mean pegvisomant dose was around 15 mg/day in all countries, except France (18.1 mg/day). At starting pegvisomant, patients treated with monotherapy ranged between 15% in the Netherlands and 72% in Spain. Monotherapy remained lowest over time in the Netherlands. In all countries, the percentage of patients with normal IGF-1 increased steeply from < 20% at baseline to 43–58% at month 6 and 51–67% at year 1. After that, we observed minor changes in the rate of acromegaly control in all countries. The Netherlands peaked in disease control at year 2 (72%). The proportion of patients reporting changes in pituitary tumor size was generally low. Serious treatment-related adverse events were < 5% in all countries.

**Conclusions:**

Our study provided a detailed summary of real-life use of pegvisomant in the six top-recruiter ACROSTUDY nations.

## Introduction

Acromegaly is a rare endocrine disease affecting 3–14 subjects per 100,000 people, according to population studies conducted in various high-income countries [1]. Its incidence was reported to range between 0.2 and 1.1 cases per 100,000 person-year. In almost all cases, this disease is due to a GH (growth hormone)-secreting pituitary adenoma leading to elevated growth hormone and insulin-like growth factor 1 (IGF-1) levels. These latter are, in turn, associated with increased mortality in acromegaly patients [2].

Various treatment options are available to treat acromegaly, including surgery, radiation therapy, and medical therapies as somatostatin receptor ligands, dopaminergic agonists, and the GH receptor antagonist. This wide therapeutic scenario has changed the natural history of acromegaly, allowing a stable biochemical control in the majority of subjects [3, 4] with a significant impact on morbidity, mortality, and quality of life [5, 6].

Pegvisomant is a growth hormone-receptor antagonist approved in the early 2000s. Its efficacy in reducing serum IGF-1 concentrations and improving clinical signs and symptoms of acromegaly was established by pivotal randomized controlled trials (RCT), both in the short- and long term [7, 8]. Subsequently, a large international surveillance study (ACROSTUDY) was started to examine pegvisomant’s safety and effectiveness in everyday clinical practice [9].

Several studies have been published during the last decade using data from both the International ACROSTUDY and its national subsets [10–16]. As a whole, these studies have confirmed the safety and efficacy of pegvisomant in the treatment of acromegaly. However, the national ACROSTUDY researches have revealed some similarities and differences between different countries in the real-world use of pegvisomant and in the clinical outcomes of the therapy [10, 11, 15].

A comprehensive picture of pegvisomant use in clinical practice in different countries is lacking. Therefore, the main aim of the study is to explore the occurrence of country-specific differences in the real-life use of pegvisomant through the ACROSTUDY data. The impact of those differences on the safety of pegvisomant and clinical outcomes of acromegaly will also be assessed.

## Materials and methods

ACROSTUDY is an open-label, non-interventional, post-marketing safety surveillance study conducted in acromegaly patients treated with pegvisomant. The study was designed to monitor the long-term safety and effectiveness in a real-world setting. Therefore, study data were collected as part of the routine clinical care of each patient. Given the observational nature of the study, all treatment-related features (i.e., dose, schedule, etc.) and the visit schedule were at the discretion of treating physicians. ACROSTUDY was conducted in compliance with the Declaration of Helsinki and with applicable local laws and requirements. Ethical approval was obtained from the Comitato Etico dell’Università Cattolica del Sacro Cuore – Policlinico Universitario Agostino Gemelli di Roma, Rome, for the Italian coordinating center (i.e., Università Cattolica del Sacro Cuore, Rome, Italy) and from local Boards or Ethical Committees for all study centers. All patients provided written informed consent before enrollment in the study.

Exclusion criteria were participation in other acromegaly trials, surgery requirements to decompress the tumor for visual field loss, cranial nerve palsies, intracranial hypertension, and rhinoliquorrhea. Detailed information on the methods of ACROSTUDY have been provided elsewhere [17, 18].

The present investigation is based on the complete analysis set of ACROSTUDY, including data from 15 countries, collected between 2004 and December 2017, when ACROSTUDY was terminated [19]. Since some nations had enrolled a limited number of patients, we focused the analysis to data from the six top-recruiter countries, i.e., Germany, Italy, France, USA, Spain and the Netherlands. Together, these nations accounted for over 85% of all ACROSTUDY cases. All acromegaly patients were already under treatment or were starting pegvisomant at enrolment in ACROSTUDY.

In ACROSTUDY, data were collected through an electronic Case Report Form (eCRF) using a web-based tool, both at baseline (using clinical records) and at each follow-up visit. For the purpose of the study, we extract information from the full ACROSTUDY database. More in detail, for each patient, we retrieved data at baseline and from each follow-up visit, as appropriate, for the following parameters: (1) socio-demographic features of the patients (e.g., age, sex, race, etc.); (2) data from physical examinations (e.g., weight, height, etc.); (3) disease-related information (e.g., date of diagnosis and symptoms, pituitary imaging, previous and concomitant therapies for acromegaly, the dose of pegvisomant); (4) presence of comorbidities (e.g., hypertension, diabetes, cardiovascular conditions, neoplasms, respiratory diseases, osteoarthritis, etc.), and v) laboratory tests (e.g., ALT, AST, serum IGF-1 levels, baseline serum GH, HbA1c, etc.).

We used the data on concomitant medications for acromegaly to stratify patients who were treated only with pegvisomant (monotherapy) or under a combined medical therapy for acromegaly (combined therapy, pegvisomant and somatostatin analogues (SSA) and/or dopamine agonists (combination therapy). All patients have been classified accordingly at baseline and yearly during their follow-up in ACROSTUDY.

All laboratory tests were conducted using commercial assays available at each study Center, and results were interpreted according to the local normal reference ranges. Serum IGF-1 values were normalized by dividing the observed value by the upper limit of the local age-adjusted normal values.

Whenever possible, pituitary MRI (magnetic resonance imaging) were conducted using the same imaging techniques and equipment in all study centers. According to the ACROSTUDY protocol, T1 weighted spin-echo (or fast spin-echo) sagittal and coronal images before and after gadolinium and T2 weighted fast spin-echo coronal images have been obtained. When the local radiologist detected a change in pituitary tumor size, all the corresponding patient images were sent for central assessment.

Safety information, including adverse events (AE), serious AE, treatment-related adverse events (TRAE), laboratory tests and MRI data, was collected and classified by study investigators using the Medical Dictionary of Regulatory Activities (MedDRA) v. 14.1. A worsening of an already existing comorbidity or condition was considered as an AE. AEs occurring after starting pegvisomant but before enrolment in ACROSTUDY, if deemed relevant, were collected in the database.

All statistical analyses in the present investigation are descriptive. Baseline data refer to the ones obtained at the start of pegvisomant treatment, independently of the time of the enrolment in ACROSTUDY. Nine years of follow-up were considered in all effectiveness analyses except for pituitary tumor size, due to the limited number of MRI available after more than 5 years of follow-up. Descriptive analyses were conducted by tabulating frequencies and percentages (for categorical variables) and range, mean, median values and standard deviations (SD, for continuous variables). Descriptive data were also examined graphically through histograms and line charts.

## Results

The baseline characteristics of patients with acromegaly enrolled in ACROSTUDY, overall and according to (major) countries, are shown in Table 1. A total of 2221 patients were enrolled, of whom 548 (24.7%) in Germany, 466 (21.0%) in Italy, 312 (14.0%) in France, 207 (9.3%) in the US, 200 (9.0%) in Spain, 175 (7.9%) in the Netherlands and 313 (14.1%) in nine other countries. The mean ± SD duration of the follow-up in ACROSTUDY was 8.5 ± 4.0 years in the Netherlands, 8.4 ± 3.2 in Germany, 7.5 ± 3.1 in Spain, 6.5 ± 3.0 in Italy, 5.9 ± 3.1 in France and 5.8 ± 3.6 in the USA. The mean age at starting pegvisomant therapy was 49.5 (SD 14.2) years, ranging between 46.6 years in France and 51.5 in the Netherlands. At the start of pegvisomant therapy, 88.4% of all patients had elevated IGF-1. The corresponding proportions in each country were 93.5% in Italy, 88.8% in France, 88.3% in Spain, 86.9% in Germany, 86.1% in the Netherlands and 83.1% in the US. The proportion of patients that have been submitted to pituitary adenomectomy before the start of pegvisomant therapy is similar in all the countries except the Netherlands where 40% of the subjects has received only medical therapy before the enrolment/start of pegvisomant. The median IGF-1 level at starting pegvisomant ranged between 348 μg/L in the Netherlands and 545 μg/L in Spain. After 1 year of follow-up, information in ACROSTUDY was available for 90.9–100.0% of patients in the six countries examined. The corresponding percentage range at 5 years of follow-up was from 48.3% to 83.5% and at 9 years from 22.4% to 28.1%.

**Table 1:** Main characteristics at baseline of patients enrolled in ACROSTUDY, overall and according to major countries

	All ACROSTUDY	Germany	Italy	France	USA	Spain	Netherlands
No. cases	2221	548	466	312	207	200	175
% males	50.8%	51.8%	49.8%	52.9%	53.6%	43.0%	55.4%
% Caucasian	92.4%	98.1%	98.5%	86.7%	84.2%	99.0%	94.1%
BMI at PEG start, mean (SD)	29.5 (5.4)	29.6 (5.1)	28.1 (5.0)	29.0 (5.4)	32.3 (7.0)	28.8 (4.6)	29.4 (4.5)
Age at diagnosis, mean (SD)	42.1 (13.6)	41.6 (13.3)	43.0 (13.7)	39.9 (13.6)	41.9 (13.6)	43.3 (13.8)	45.4 (13.9)
Age at PEG start, mean (SD)	49.5 (14.2)	49.7 (14.4)	51.1 (14.0)	46.6 (14.3)	46.8 (14.1)	49.6 (14.2)	51.5 (13.4)
Years of PEG therapy, mean (SD)	8.5 (4.4)	9.3 (4.3)	6.8 (4.0)	8.2 (4.6)	7.2 (4.7)	11.0 (3.1)	10.1 (3.9)
Age at ACROSTUDY entry, mean (SD)	51.0 (14.3)	51.1 (14.5)	52.5 (13.9)	48.4 (14.4)	48.2 (14.3)	51.7 (14.4)	53.3 (13.5)
Years in ACROSTUDY, mean (SD)	7.2 (3.4)	8.4 (3.2)	6.5 (3.0)	5.9 (3.1)	5.8 (3.6)	7.5 (3.1)	8.5 (4.0)
% on PEG therapy since more than 1 year at ACROSTUDY entry	46.3%	44.8%	39.2%	56.7%	35.7%	69.0%	56.7%
Elevated IGF-1 at diagnosis	82.9%	81.2%	82.6%	86.9%	87.4%	84.5%	84.6%
IGF-1 at diagnosis, median (p10, p90)*	842 (361, 1500)	771 (292, 1175)	790 (447, 1258)	960 (464, 1700)	675 (294, 1458)	842 (438, 1474)	667 (280, 1439)
Elevated IGF-1 at PEG start	88.4%	86.9%	93.5%	88.8%	83.1%	88.3%	86.1%
IGF-1 at PEG start, median (p10, p90)	461 (228, 945)	434 (219, 889)	461 (242, 924)	533 (277, 1230)	443 (196, 943)	545 (258, 1056)	348 (148, 757)
Biochemical Diagnosis of GH hypersecretion (%)	93.7%	92.2%	91.0%	95.8%	94.2%	97.0%	97.1%
Failure to suppress GH at OGTT (%)	56.6%	66.8%	57.9%	41.7%	30.0%	62.0%	65.7%
% pituitary microadenoma	7.6%	5.3%	11.2%	5.8%	7.7%	2.5%	11.4%
% pituitary macroadenoma	42.5%	37.0%	50.2%	52.9%	47.8%	23.0%	37.1%
Treatment before PEG start							
Any treatment	96.3%	95.1%	97.6%	97.8%	93.7%	96.5%	95.4%
Medical therapy only	18.8%	11.9%	25.3%	18.9%	9.2%	15.0%	40.0%
Radiation only	0.0%	0.0%	0.0%	0.0%	0.0%	0.0%	0.6%
Surgery only	4.1%	3.3%	2.1%	1.9%	15.0%	3.0%	2.3%
Medical and radiation	2.0%	1.6%	1.1%	2.6%	1.0%	0.5%	0.0%
Medical and surgery	48.1%	55.1%	54.7%	50.3%	42.5%	44.0%	28.6%
Radiation and surgery	1.6%	1.3%	0.0%	0.0%	9.2%	2.0%	1.7%
Medical, radiation and surgery	21.6%	21.9%	14.4%	24.0%	16.9%	32.0%	22.3%

*BMI* body mass index, *PEG* pegvisomant, *IGF-1* insulin-like growth factor 1, *GH* growth hormone, *OGTT* oral glucose tolerance test

*IGF-1 data collected within 12 months from diagnosis

**Fig. 1:**
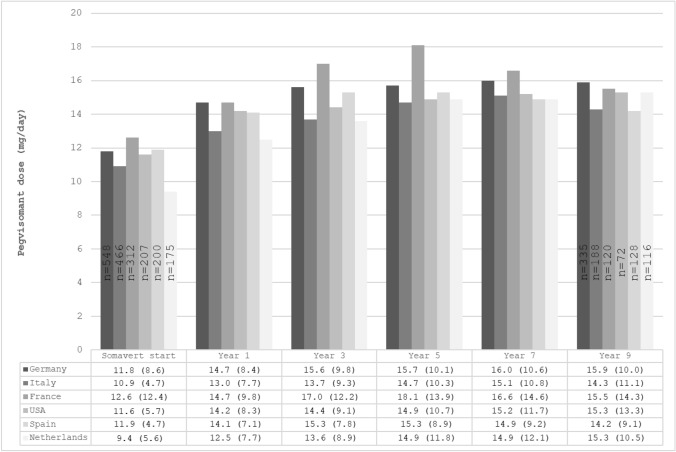
Mean daily administered dose of pegvisomant over time in six countries included in ACROSTUDY. Data in the bars indicate the number of patients with information on pegvisomant dose at treatment start and at year 9. Data below the bars are means (SD)

Figure 1 shows the mean daily treatment dose at pegvisomant therapy start and after years 1, 3, 5, 7 and 9 in patients with acromegaly enrolled in the six top-recruiter countries. The mean dose at treatment start was lowest in the Netherlands (9.4 mg/day), with other countries reporting mean dosages between 10.9 mg/day (Italy) and 12.6 mg/day (France). Pegvisomant mean daily dose increased from baseline up to year 3 in Germany and Spain and up to year 5 in other countries, then tended to level off. In year 5, most countries reported a mean pegvisomant dose of around 15 mg/day, except France, where we observed an increment of mean daily dose (18.1 mg/day). At year 9, the mean pegvisomant dose was between 14 and 16 mg/day in all included countries.

**Fig. 2:**
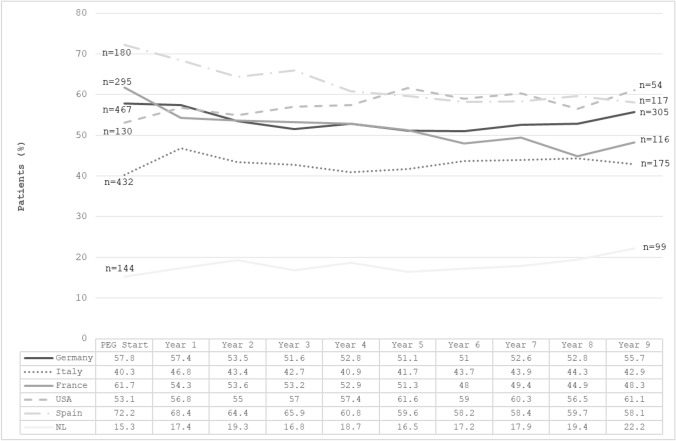
Proportion of acromegaly patients treated with pegvisomant alone over time in six countries included in ACROSTUDY. Data besides the lines indicate the number of patients with information on pegvisomant use alone or in combination at treatment start and at year 9

The proportion of acromegaly patients treated with pegvisomant alone at baseline during the follow-up period is reported in Fig. 2. Differences in mono- vs. combined therapy emerged between countries at starting pegvisomant: patients treated with pegvisomant alone were 15.3% in the Netherlands, 40.3% in Italy, 53.1% in the USA, 57.8% in Germany, 61.7% in France and 72.2% in Spain. The proportion of patients treated with pegvisomant alone was lowest over the whole period in the Netherlands, showing modest (increasing) variations up to year 9 (22.2%). Similarly, pegvisomant monotherapy use did not show major changes during follow-up in Italy (ranging between 40.3% and 46.8% of patients) and in Germany (between 51.0% and 57.8%). We observe a tendency to switch from mono- to combined therapy over time in Spain (with monotherapy decreasing from 72.2% at baseline to < 60% from year 5 onwards) and in France (from 61.7% at baseline to < 50% after year 5). Conversely, pegvisomant monotherapy increased slightly in the USA, from 53.1% at baseline to 61.6% at year 5 and 61.1% at year 9. Among patients treated with combination therapy, somatostatin analogues (SSAs) were the most frequent drug associated with pegvisomant in all countries, both at baseline and during follow-up (data not shown).

**Fig. 3:**
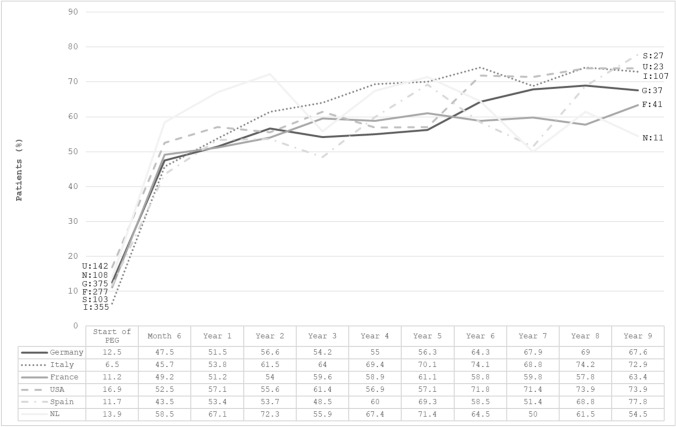
Proportion of acromegaly patients with normal IGF-1 over time in six countries included in ACROSTUDY. Data besides the lines indicate the number of patients with information on normal IGF-1 (by country, identified by the first letter) at treatment start and at year 9

Figure 3 shows the proportion of acromegaly patients with normal IGF-1 at baseline and during follow-up in the six nations included in our survey. In all countries, the percentage of patients with normal IGF-1 increased rapidly from < 20% at baseline to 43–58% at month 6 and 51–67% at year 1. After 5 years, the proportion of patients with normal IGF-1 was around 55–60% in three countries (Germany, USA and France) and about 70% in all the others (Spain, Italy and the Netherlands). The Netherlands had the highest proportion of patients with normal IGF-1 during the first 2 years of follow-up. At the last available visit, the proportion of patients with normal IGF-1 ranked between 59% in France, 60% in Germany, 62% in the USA, 64% in Spain and the Netherlands and 68% in Italy (data not shown).

**Fig. 4:**
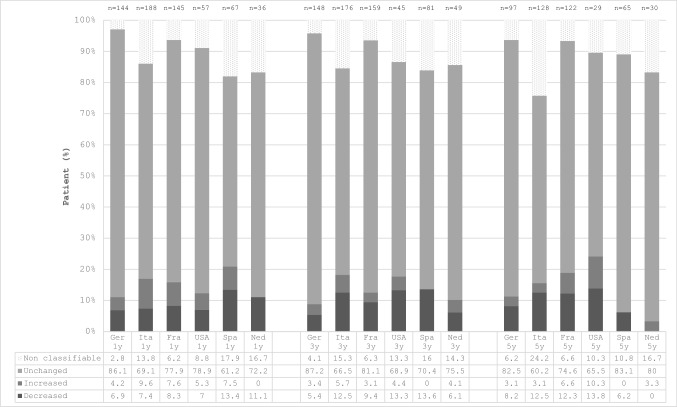
Proportions of acromegaly patients with change from baseline in pituitary tumor size over time (at 1 year, 3 years and 5 years) in six countries included in ACROSTUDY. Data above the bars indicate the number of patients with available MRI image change from baseline at each time period. y: year

Figure 4 illustrates the changes from baseline in pituitary tumor size of acromegaly patients. Most patients reported no change in pituitary tumor size. Between 6.9% (Germany) and 13.4% (Spain) of patients reported a decreased pituitary tumor size after 1 year from the enrollment in ACROSTUDY. Results were generally similar—though with some differences between countries—at year 5. The proportion of patients with increased pituitary tumor size was generally low in all countries and at all time points.

Table 2 summarizes AE and TRAE occurring in patients enrolled in ACROSTUDY, stratified according to countries. A higher frequency of AE was reported in France (in 92.0% of patients) than in other countries (between 46.9% and 57.5%). Serious AE occurred in 33.0% of patients in France, 31.2% in Germany, 20.3% in the USA, 18.0% in Spain, 17.4% in Italy and 17.1% in the Netherlands. TRAE were also more frequently reported in France (41.7% vs. 8.0% to 17.9% in other five countries), whereas severe TRAE were below 5% in all countries: 4.4% in Germany, 3.9% in the USA, 1.5% in Spain, 1.3% in France and 0.6% in Italy and the Netherlands.

**Table 2:** Adverse events occurring in patients enrolled in different countries of the ACROSTUDY

	Germany	Italy	France	USA	Spain	Netherlands
Subjects evaluable for adverse events	548	466	312	207	200	175
Any adverse event						
Total number of adverse events	859	821	2338	630	216	278
Subjects with adverse events, *n* (%)	294 (53.6)	240 (51.5)	287 (92.0)	119 (57.5)	97 (48.5)	82 (46.9)
Subjects with serious adverse events, *n* (%)	171 (31.2)	81 (17.4)	103 (33.0)	42 (20.3)	36 (18.0)	30 (17.1)
Subjects with dose reduced due to serious adverse events, *n* (%)	1 (0.2)	0 (0.0)	2 (0.6)	0 (0.0)	1 (0.5)	0 (0.0)
Subjects with drug withdrawn due to serious adverse events, *n* (%)^a^	43 (7.8)	31 (6.7)	28 (9.0)	17 (8.2)	18 (9.0)	11 (6.3)
Subjects discontinued treatment due to death, *n* (%)	23 (4.2)	19 (4.1)	13 (4.2)	4 (1.9)	11 (5.5)	8 (4.6)
Treatment-related adverse events (TRAE)						
Total number of TRAE	119	70	239	72	34	28
Subjects with TRAE, *n* (%)	72 (13.1)	53 (11.4)	130 (41.7)	37 (17.9)	23 (11.5)	14 (8.0)
Subjects with serious TRAE, *n* (%)	24 (4.4)	3 (0.6)	4 (1.3)	8 (3.9)	3 (1.5)	1 (0.6)
Subjects with dose reduced due to serious TRAE, *n* (%)	0 (0.0)	0 (0.0)	1 (0.3)	0 (0.0)	0 (0.0)	0 (0.0)
Subjects with drug withdrawn due to serious TRAE, *n* (%)^a^	9 (1.6)	3 (0.6)	1 (0.3)	6 (2.9)	2 (1.0)	1 (0.6)
Subjects discontinued treatment due death, *n* (%)	0 (0.0)	0 (0.0)	0 (0.0)	0 (0.0)	0 (0.0)	0 (0.0)

^a^Drug withdrawn can be temporarily, permanently or dose delayed

## Discussion

This study provides a comprehensive description of the clinical use as well as of the safety and effectiveness of pegvisomant in the six top-recruiter countries of ACROSTUDY. Relevant differences in the use of pegvisomant emerged between countries, particularly for the Netherlands where the initial dose was lowest, and pegvisomant therapy was mainly combined with SSAs and/or other drugs. The effectiveness of treatment, measured by the proportion of patients achieving normal IGF-1, was generally consistent with the results of a recent meta-analysis [20]. The proportion of patients with normal IGF-1 was above 50% in all countries 1 year after starting the treatment and remained high afterwards. The Netherlands showed the highest peak in the percentage of patients with normal IGF-1 during the first 2 years of pegvisomant treatment, reaching 72.3% at year 2. Overall, a mean dose of around 15 mg/day (whether in mono or combination treatment setting) across the observed countries could indicate an inadequate dose optimization when considering the higher efficacy rates of over 90% IGF-1 normalization observed in registration trials. Pituitary tumor size showed minor variations in all countries subset of patients. These tended to occur during the first year of treatment and remained generally stable after that. AE and TRAE were reported more frequently in France than in the other five countries. However, serious TRAE were similar between countries, thus suggesting some heterogeneity in the accuracy of reporting of (non-serious) AE in ACROSTUDY in different nations.

The treatment approach of acromegaly with pegvisomant showed some variations between countries. In particular, in the Netherlands, pegvisomant was used predominantly in combination with SSA and—possibly because combination treatment may require a lower pegvisomant dose [21]—its initial mean dose was also about 20% lower than in other examined countries. However, it is known that over time, in both controlled and uncontrolled patients, it is possible to observe the need for an increase in the daily pegvisomant amount: this phenomenon occurs in both pegvisomant combination and monotherapy treatment, without dose differences [22, 23]. The distinct treatment patterns observed between nations may at least in part be explained by differences of acromegaly cases at baseline between countries, e.g., in IGF-1 level at baseline, as well as by a different treatment history before pegvisomant start. In fact, in the Netherlands, the median IGF-1 level at baseline was lowest, and it was previously shown that the pegvisomant dose at treatment start and during the first months/years of therapy is proportional to IGF-I levels before starting treatment [10, 15, 24]. Further, medical therapy alone was reported by 40% of patients in the Netherlands, as compared to 10–25% in other countries where, instead, a combination of surgery and medical therapy was most frequent. At subsequent yearly visits, the pegvisomant dose used in the Netherlands tended to align with other countries. At the same time, the frequency of combined therapy remained largely higher than in the rest of the EU and the USA. The different strategy of pegvisomant use in the Netherlands is apparently reflected in an increased effectiveness to control the disease, particularly during the first years of treatment. Results that might be due at an initial selection of cases, based on the severity of the patient clinical situation. Given the descriptive nature of this analysis, however, it is not in our scope to determine whether such a potential effect is real. Other relevant differences at baseline emerged. In particular, failure to suppress GH at OGTT, an important test for the diagnosis of acromegaly, varied widely between countries, with the lowest proportions reported in the USA (30%) and France (42%). This may be explained by the fact that in some patients: (1) OGTT could not be performed for concomitant diabetes; (2) GH and IGF-1 levels could be so high that GH suppression test is not compulsory; (3) data could be not available.

The proportion of patients treated with monotherapy at baseline and during the observation period showed a relevant variability. In some countries, namely Spain, France and—to a lower extent—Germany, the proportion of patients treated with monotherapy tended to decrease during the follow-up. Our result agrees with a previous report showing that the use of combined therapy increases over time [23]. Conversely, in Italy and the Netherlands, which had low baseline proportions of pegvisomant monotherapy, these remained generally stable over time. A slight increase in monotherapy use was reported in the USA.

Our data may reflect the differences in pegvisomant indications between the EU and the USA. In Europe, pegvisomant is approved in patients with inadequate response to surgery and/or radiation and failing SSAs (i.e., second-line treatment). On the contrary, in the USA pegvisomant has been approved for patients with inadequate response to any of these three approaches [25]. Further, some heterogeneity in selected patient characteristics, with a potential influence on treatment choices, emerged between US and European countries. For example, a higher BMI (Body Mass Index) at baseline was reported in the USA (mean BMI: 32.3 kg/m^2^), and obese patients may require higher doses of pegvisomant [11, 26]. However, these differences are not obviously reflected in the treatment approach used, nor in the findings on the effectiveness to control disease, as these characteristics and results are—except for the trends in monotherapy use described above—broadly in agreement between the USA and most EU countries. Similarly, women were found to require higher doses of pegvisomant than men to normalize IGF-1 [27]. The proportion of enrolled women was somewhat higher in the Spanish ACROSTUDY (57%, vs. 49% overall), but no relevant difference in pegvisomant dose in Spain was found.

With further reference to clinical endpoints, a few variations from baseline were reported in pituitary tumor size in all the examined national ACROSTUDY databases. Interestingly, many of those variations occurred during the first year of observation after starting pegvisomant treatment. After that, the treatment with pegvisomant seems to be associated with substantial stability of pituitary lesion in almost all patients. This information can therefore be useful to physicians to optimize the timing of MRI examinations.

The differences observed in the occurrence of AE between France and other nations are challenging to interpret. Since no significant differences between countries emerged according to severe TRAE and drug discontinuations due to serious AE or death, we hypothesize a different attention and precision (or perhaps an over-interpretation) in reporting non-serious AE in French investigators in comparison to other countries. As this is an observational study conducted in a real-life clinical practice setting, rather than an RCT with strict definitions and reporting rules, some variations were expected. A favorable safety profile of pegvisomant [20] was, in any case, confirmed in all countries examined.

Our investigation has several limitations typical to observational studies and particularly to analyses descriptive in nature. Since the analysis had no inferential aim, no statistical tests for comparison between countries were performed, nor analyses adjusted for potentially relevant covariates (e.g., IGF-1 at baseline) were conducted. Still, this report may be useful to generate hypotheses and to plan new analyses on acromegaly treatment. Selection bias of patients may have occurred, mainly due to two issues: first, at baseline, a selection towards inclusion of patients with more serious acromegaly may stem from pegvisomant indication of use in those who had failed earlier treatment(s); this is confirmed by ACROSTUDY data, similar across countries, showing that over 90% of patients had at least one medical treatment before starting pegvisomant. Second, during follow-up, a selection of patients with more favorable outcomes may have occurred in this long-term, prospective study. For the latter, the results reported during the first years of follow-up (e.g., up to 5 years) should, however, not be considerably affected by this issue. The representativeness of data, i.e., which proportion of patients treated with pegvisomant was enrolled, was not strictly recorded in each country. In the German cohort, however, more than 80% of all patients with pegvisomant prescriptions in 2004–2008 had been enrolled in the study [16]. Real-world studies lack the rigorous methodology of randomized trials. Still, they have the strength to provide information in unselected populations of routinely treated patients, and thus contribute providing data on the treatment use, and on their safety and effectiveness, in the real-life setting [28].

In conclusion, we have explored the pegvisomant therapeutic behavior in the six top-recruiter countries of ACROSTUDY. This study provides an overview of the similarities and differences in the use of pegvisomant among these nations, as well as the impact of these variations in the safety profile and effectiveness of pegvisomant. An optimization of pegvisomant use in the management of acromegaly is still needed [29]. This analysis describing the treatment schemes used across nations may provide valuable insights to clinicians.
